# Genetic Variation and Trait Correlations in an East African Cassava Breeding Population for Genomic Selection

**DOI:** 10.2135/cropsci2018.01.0060

**Published:** 2019-01-24

**Authors:** Alfred Ozimati, Robert Kawuki, Williams Esuma, Siraj I Kayondo, Anthony Pariyo, Marnin Wolfe, Jean-Luc Jannink

**Affiliations:** 1 A. Ozimati, R. Kawuki, W. Esuma, S.I. Kayondo, and A. Pariyo, National Crops Resources Research Institute (NaCRRI), PO Box, 7084 Kampala, Uganda; 2 A. Ozimati, M. Wolfe, and J.-L. Jannink, School of Integrative Plant Science, Plant Breeding and Genetics Section, Cornell Univ., Ithaca, NY, 14853; 3 M. Wolfe and J.-L. Jannink, USDA-ARS, R.W. Holley Center for Agriculture and Health, Ithaca, NY 14853

## Abstract

Cassava (*Manihot esculenta* Crantz) is a major source of dietary carbohydrates for >700 million people globally. However, its long breeding cycle has slowed the rate of genetic gain for target traits. This study aimed to asses genetic variation, the level of inbreeding, and trait correlations in genomic selection breeding cycles. We used phenotypic and genotypic data from the National Crops Resources Research Institute (NaCRRI) foundation population (Cycle 0, C_0_) and the progeny (Cycle 1, C_1_) derived from crosses of 100 selected C0 clones as progenitors, both to evaluate and optimize genomic selection. The highest broad-sense heritability (*H*^2^ = 0.95) and narrow-sense heritability (*h*^2^ = 0.81) were recorded for cassava mosaic disease severity and the lowest for root weight per plot (*H*^2^ = 0.06 and *h*^2^ = 0.00). We observed the highest genetic correlation (*r*_g_= 0.80) between cassava brown streak disease root incidence measured at seedling and clonal stages of evaluation, suggesting the usefulness of seedling data in predicting clonal performance for cassava brown streak root necrosis. Similarly, high genetic correlations were observed between cassava brown streak disease severity (*r*_g_= 0.83) scored at 3 and 6 mo after planting (MAP) and cassava mosaic disease, scored at 3 and 6 MAP (*r*_g_= 0.95), indicating that data obtained on these two diseases at 6 MAP would suffice. Population differentiation between C_0_ and C_1_ was not well defined, implying that the 100 selected progenitors of C_1_ captured the diversity in the C_0_. Overall, genetic gain for most traits were observed from C_0_ to C_1_.

CASSAVA (*Manihot esculenta* Crantz) is a crop that provides staple food for >700 million people worldwide (Edgerton, 2009; Burns et al., 2010). Although cassava was domesticated >6000 yr ago (Olsen and Schaal, 1999), it has had only a short exposure to formal breeding compared with other staple crops, such as maize (*Zea mays* L.), rice (*Oryza sativa* L.), and wheat (*Triticum aestivum* L.) (Ceballos et al., 2004; Fischer and Edmeades, 2010). Formal cassava breeding in Africa only began in the 1930s at the Amani Research Station in Tanzania, where efforts were made to combat epidemics of cassava mosaic disease (CMD) and cassava brown streak disease (CBSD) (Storey and Nichols, 1938). Since then, breeding efforts have yielded substantial genetic improvement in cassava for agronomic traits, including CMD (Jennings and Iglesias, 2002).

However, CBSD has remained a major limitation to cassava production in eastern, central, and southern Africa (Alicai et al., 2007; Hillocks and Maruthi, 2015), with the lack of resistant varieties amplifying the geographical spread of the disease. The rapid growth of the human population in sub-Saharan Africa and the escalating effects of climate change justify the need for accelerating the rate of genetic gain to increase the productivity of cassava (Burns et al., 2010).

The most commonly used method for breeding cassava remains phenotypic recurrent selection, which requires 8 to 10 yr of evaluation prior to official cultivar release and selection of parents for the next cycle of recombination (Ceballos et al., 2016). The long breeding cycle makes it challenging for breeders to respond quickly to farmers’ needs of high-yielding and disease-resistant cultivars. Fortunately, the availability of relatively cheap next-generation sequencing technologies, such as genotyping-by-sequencing (GBS), has made it possible to profile single nucleotide polymorphic (SNP) markers across a genome (Elshire et al., 2011). This technology enables mapping of quantitative trait loci (QTL) and application of genome-wide predictions, as proposed by Meuwissen et al. (2001).

Genomic selection (GS) involves the prediction of breeding values and selection of parents based on marker-estimated effects, enabling more cycles of selection and recombination per unit time than phenotypic recurrent selection (Bhat et al., 2016). Thus, GS will potentially shorten the breeding cycle of cassava and enable breeders to meet the growing need for improved varieties. However, for GS to be successfully applied in breeding, a number of factors must be considered, including the level of genetic variability and the heritability of the traits for which genome-wide predictions are targeted (Jannink et al., 2010; Muranty et al., 2015).

The National Crops Resources Research Institute (NaCRRI) of Uganda is one of the first cassava breeding programs in Africa to implement GS. Genomic prediction accuracies from the initial training population (C0) at NaCRRI have been estimated to be reasonably accurate for highly heritable traits, such as dry matter content (DMC) and CMD, but less for low heritability traits, such as fresh root yield (Wolfe et al., 2017). Within the same population (C0), mean prediction accuracies for CBSD-related traits spanned from 0.24 to 0.43 for CBSD foliar symptoms, and from 0.32 to 0.45 for CBSD root necrosis across a number of tested genomic prediction models (Kayondo et al., 2018). Whereas Wolfe et al. (2017) focused on predicting yield traits and CMD, which are common problems across all cassava breeding programs in Africa, Kayondo et al. (2018) specifically focused on QTL mapping and genomic predictions for CBSD-related traits, a problem facing cassava production only in eastern, central, and southern parts of Africa.

In cassava breeding, one of the potential benefits of GS is that selections can be made at the seedling stage, especially for highly heritable traits, for subsequent crossing. Selections at the seedling stage would offer the advantage of reducing the breeding cycle, especially when the correlation between clonal and seedling performance for the target trait(s) is high. At the International Center for Tropical Agriculture (CIAT), the 8-yr cassava breeding cycle was reportedly shortened to only 3 yr, when parental selections for subsequent crossing were made at the seedling stage for total carotenoid content (Ceballos et al., 2013). Genetic correlations between seedling and clonal trait expressions have not yet been ascertained in our breeding population; therefore, one of our objectives was to estimate the genetic correlations between seedling and clonal evaluated traits.

Cassava breeding requires selecting for multiple traits to enhance cultivar adoption rate (Barandica et al., 2016). Multi-trait breeding goals are easier to achieve when favorable genetic relationships, arising from linkage or pleiotropy, exist among target traits (Lynch and Walsh, 1998). Phenotypic correlations could be attributed to genetic effects, a common environment, or error deviations. On the other hand, an additive genetic correlation between any two traits implies a relationship between the breeding values of individuals (Bernardo, 2003). In cassava, undesirable phenotypic and genotypic correlations have been reported for some important traits (Njoku et al., 2015; Barandica et al., 2016; Esuma et al., 2016). For example, an undesirable negative genetic correlation (*r*_g_ = −0.45) between DMC and total carotenoid content has been observed in African cassava breeding population (Esuma et al., 2016). In addition to seedling–clonal genetic correlations, it was also of interest to investigate the genetic correlation among clonal evaluated traits.

Furthermore, cassava is known to suffer from inbreeding depression (Rojas et al., 2009; Kaweesi et al., 2014; Ramu et al., 2017). With rapid GS, there is a risk to exacerbate the inbreeding, mainly because of increased selection intensity per unit time. The impact that GS will have on inbreeding in cassava is not yet known, particularly for the NaCRRI GS program. The NaCRRI has recently completed its first cycle (C_1_) of GS, including seedling and clonal phenotypic evaluations of a large portion of the C_1_ population. This presents the opportunity to address a number of unanswered questions and assess the progress made so far relative to GS in this population, using both the available C_0_ and C_1_ datasets. Thus, our overall objective was to estimate genetic parameters to guide routine implementation of GS in an East African cassava breeding population. Our specific objectives were (i) to assess trait variability, genetic diversity, and inbreeding level in C_0_ and C_1_ genotypes that constitute the GS training population at NaCRRI, Uganda; and (ii) to examine the phenotypic and genetic correlations for selected agronomic and virus resistance traits evaluated at the seedling and clonal stages.

## MATERIALS AND METHODS

### Constitution of C_0_ and C_1_ Populations

In response to the CBSD outbreak in Uganda (Alicai et al., 2007), an initiative was undertaken in 2009 to assemble sources of resistance to facilitate the development of breeding populations for genetic improvement and subsequent on-farm deployment. Accordingly, germplasm was introduced from CIAT, International Institute of Tropical Agriculture (IITA), and Tanzania’s national research program. Germplasm from Tanzania was received as botanical seed, whereas materials from CIAT and IITA were introduced as tissue culture plantlets. Hybridizations were made among 52 parents introduced between 2009 and 2010, using a partial diallel mating design (Supplemental Table S1). From the progenies generated (full-sibs and half-sibs), 395 clones were selected in 2012 and 2013 to constitute a base population (C_0_) for GS. A subset of 100 C_0_ clones was selected for hybridization to produce the C_1_ population.

In order to select progenitors to generate C_1_, we used a selection index (SI). Our selection index included four traits, which collectively represent the major breeding objectives of our program: CBSD root severity (CBSDRs), DMC, harvest index (HI), and root weight per plot (RTWT). As indicated above, our breeding program is implementing GS. We derive genomic estimated breeding values (GEBVs) for each of the traits mentioned using mixed-model methods described in detail below. Since our selection criteria are already estimates of breeding value, it was not necessary to further account for the difference between phenotypic and genetic variance-covariance (Ceron-Rojas et al., 2015) in constructing our selection index. Instead, we simply mean centered and variance standardized the GEBVs for each of the four traits and applied the following formula:

$$\text{SI}=1\left(\text{DMC}\right)+1\left(\text{HI}\right)+1\left(\text{RTWT}\right)-2\left(\text{CBSDRs}\right)$$

The weight of −2 was used for CBSDRs as a positive value of the GEBV to indicate worse-than-average disease symptoms.

For genotyping, DNA was extracted from ~100 mg of fresh young leaves from each of the C_0_ clones. All extractions were done using a QIAGEN DNeasy extraction kit, and DNA was quantified to ensure the required concentrations for sequencing were obtained. The DNA samples were genotyped using the GBS method described by Elshire et al. (2011). Details of the SNP calling, filtering, and imputation pipeline we used have been provided previously (Hamblin and Rabbi, 2014; Wolfe et al., 2016, 2017). Furthermore, the C_0_ clones selected to be parents of C_1_ were grouped into four clusters, using *K*-means clustering (Lloyd, 1982), implemented on the realized genomic relationship matrix, which was constructed from GBS SNP markers. During crossing of selected parents, priority was given to between-cluster rather than within-cluster crosses to reduce the risk of inbreeding. Hybridizations and seed handling were conducted using the standard procedure described by Kawano (1980) and Mezzalira et al. (2013).

### Field Evaluation of the C_0_ Population

In April 2013, a panel of 395 C_0_ clones (herein referred to as the training population) was planted at three locations: Namulonge in central Uganda (0. 311799° N, 32. 363239° E), Ngetta in northern Uganda (2.14500° N, 32.54000° E), and Kasese in southwestern Uganda (0.105999° N, 30.45999° E) to assess their agronomic performance and reaction to CMD and CBSD. Importantly, Namulonge is known to be a hotspot for CMD and CBSD (Kaweesi et al., 2014; Pariyo et al., 2015). At each location, single-row plots of 10 plants were established in a 33 ´ 13 a-lattice design, with 33 incomplete blocks and two replications. Plant spacing of 1 ´ 1 m was adopted within and between rows, whereas blocks were separated by 2-m alleys. No fertilizers were applied during the course of the experiment. Weeding was done manually whenever necessary, and the experiments were entirely rainfed.

At 3 and 6 mo after planting (MAP), all plants were assessed for CMD and CBSD shoot symptoms, whereas CBSD root necrosis severity was scored at harvest (12 MAP). Shoot severity for CBSD was assessed on a scale of 1 to 5 (Hillocks and Thresh, 2000), where 1 = no symptoms; 2 = slight foliar chlorotic leaf mottle with no stem lesions; 3 = foliar chlorotic leaf mottle and blotches with mild stem lesions, but no dieback; 4 = foliar chlorotic leaf mottle and blotches with pronounced stem lesions, but no dieback; and 5 = defoliation with stem lesions and dieback. Foliar incidence for CBSD was computed as a percentage of symptomatic plants per plot. At harvest, all roots in a plot were pooled and assessed individually for CBSD necrosis. Each root was cut transversely into five to seven pieces, and the cross-sections were scored for necrotic symptoms on a scale of 1 to 5 (Hillocks and Thresh, 2000), where 1 = no necrosis, 2 = £5% necrotic; 3 = 6 to 10% necrotic; 4 = 11 to 25% necrotic and mild root constriction; and 5 = >25% necrotic and severe root constriction. Root incidence for CBSD was computed as a percentage of necrotic roots per plot. Similarly, CMD severity was scored on a scale of 1 to 5 (IITA, 1990), where 1 = no symptoms; 2 = mild chlorotic pattern across the entire leaf, although the leaf appears green and healthy; 3 = moderate mosaic pattern throughout the leaf, narrowing and distortion in the lower one-third of leaflets; 4 = severe mosaic, distortion in two-thirds of the leaflets, and general reduction in leaf size; and 5 = severe mosaic distortion in the entire leaf. Foliar incidence for CMD was computed as a percentage of symptomatic plants per plot. On the other hand, plant vigor was evaluated at 3 MAP on an ordinal scale of 3 to 7, where 3 = low vigor, 5 = moderate vigor, and 7 = high vigor. At harvest, the aboveground biomass and storage roots for each plot were weighed separately. Harvest index was computed as a ratio of root weight to total biomass. To measure DMC, 2 to 5 kg of roots was weighed in air and in water to enable computation of specific gravity, which was subsequently used to estimate DMC, as described by Kawano et al. (1987) as follows:

$$\text{DMC}=\left[158.3\left(\frac{W_{a}}{W_{a}-W_{W}}\right)\right]-142$$

where *W*_a_ and *W*_w_ represent weights in air and water, respectively. The ratio in the formula is the specific gravity of the roots. The numbers 158.3 and 142 are the regression coefficient and the intercept, respectively, which were empirically determined by Kawano et al. (1987).

### Field Evaluation and Genotyping of the C_1_ Population

The C_1_ seeds generated from crosses among the top 100 progenitors selected from C_0_ were processed and germinated under controlled screen house conditions (Mezzalira et al., 2013), and the resultant 4874 C_1_ seedlings were transplanted at Namulonge for field evaluation in May 2015. Seedlings were assessed for shoot CMD and CBSD severity at 3 and 6 MAP. At 15 MAP (August 2016), plants were harvested and CBSD root necrotic symptoms were assessed, as described above. We did not have budget to genotype all C_1_ seedlings. For this reason, we decided to cull plants with CMD severity score of 3 or greater, as well as those with insufficient stem biomass to generate at least 10 cuttings for subsequent clonal evaluations. We made this decision because CMD is a high-heritability trait and is easily scored on seedlings. Because we did not cull on the basis of any other trait, the only bias in selection response should be for CMD. Prior to harvesting, leaf samples were collected from 2113 selected C_1_ seedlings for DNA extraction, as described above. Of the 2113 seedlings, 1420 were cloned and evaluated at Namulonge. A subset (1088) of the clones established at Namulonge was evaluated at Serere in eastern Uganda (1.295999° N, 33.325999° E) to capture further variability that might be associated with environmental differences. The clonal trials were established in August 2016, using an augmented design comprising 30 to 34 plots per block. Each clone was represented by a row of 10 plants and each block contained four checks. Assessment for CMD, CBSD, DMC, and HI was done, as described for C_0_ evaluations. Because of missing plots, a total of 1056 C_1_ clones remained for downstream data analyses, of which 432 and 624 were full-sib and half-sib progenies, respectively. Similarly, the top 110 clones were selected from C_1_ population as parents to generate C_2_ using the selection index described above, and the parents were clustered using SNP markers, as described previously.

### Statistical Analyses

To enable estimation of genetic variance and further compute broad-sense heritability for traits measured in C_0_ and C_1_ clonal evaluations, phenotypic data from the two sets of experiments were fitted to a linear mixed model using the *lme4* package for the R statistical computing software (R Development Core Team, 2008). For analysis of C_0_ phenotypic data, the following model was fitted:

$$y=X\beta +Z_{clone}c+Z_{block\left(rep\right)}b+e$$

where **β** is a vector of fixed effects of locations and grand mean, and **X** is the incidence matrix linking observations to those effects; vector **c** is a random effect for clones, where $c\sim N\left(0,I\sigma_{c}^{2}\right)$, **Z_clone_** is the corresponding incidence matrix, and **I** is the identity matrix; vector **b** is a random effect for blocks nested in replication, such that $b\sim N\left(0,I\sigma_{b}^{2}\right)$, and **Z_block(rep)_** is the corresponding incidence matrix; and **e** is the residual, such that $e\sim N\left(0,I\sigma_{e}^{2}\right)$. Variance components were extracted from the model used to compute broad-sense heritability (*H*^2^) estimates as follows:

$$H^{2}=\sigma_{c}^{2}/\left(\sigma_{c}^{2}+\sigma_{e}^{2}\right)$$

where $\sigma_{c}^{2}$ is clone variance and $\sigma_{e}^{2}$ is model residual variance. Similarly, we fitted a mixed model for the C_1_ trial, including a fixed effect of location and grand mean, a random effect for clones, blocks nested in location, and the random residual term. Accordingly, variance components were extracted to compute broad-sense heritability estimates for C_1_ clones.

In addition, we fitted a single-step genomic best linear unbiased predication (G-BLUP) model, first for C_0_ and C_1_ populations separately, and later we combined the two populations for joint analysis. From separate analyses, we estimated SNP-based heritability (narrow-sense heritability) for all traits, using the formula as described below:

$$h^{2}=\sigma_{a}^{2}/\left(\sigma_{a}^{2}+\sigma_{e}^{2}\right)$$

where $\sigma_{a}^{2}$ is the additive genetic variance, *h*^2^ is the narrow-sense heritability, and $\sigma_{e}^{2}$ is the model residual variance.

For the combined C_0_ and C_1_ data, we fitted a mixed model where the trial location and grand mean were treated as fixed effects, and clone effects were considered random, with realized genomic relationship matrix **K** constructed using the *A.mat* function in the rrBLUP package for the markers (Endelman, 2011). The GEBVs were extracted for various traits from the G-BLUP model for combined datasets analyzed and averaged for each population (C_0_ and C_1_). Furthermore, we performed a *t* test to compare mean differences in the GEBVs between C_0_ and C_1_ populations. Boxplots were generated from the GEBVs, using the *ggplot* function built in ggplot2 in R to visualize variability for each trait between the two populations.

Both phenotypic and genetic correlations were estimated for three scenarios: (i) among C_1_ traits evaluated at the seedling stage, (ii) between C_1_ traits measured at the seedling and at clonal stages, and (iii) combined data for C_0_ and C_1_ traits evaluated at the clonal stage. To estimate the phenotypic correlations, we used the raw data without accounting for the field trial designs. For estimation of genetic correlations, we first fitted multi-locational models described above for C_0_ and C_1_ datasets separately. From linear mixed models, best linear unbiased predictors (BLUPs) were extracted for both C_0_ and C_1_ clones and deregressed using the formula described by Garrick et al. (2009).

The genomic breeding values were estimated using multivariate G-BLUP with the *emmremlMultivariate* function in the EMMREML package (Akdemir and Okeke, 2015) in R. As only a single observation on each seedling was recorded, a single-step genomic mixed-model was appropriate to fit seedling data for estimation of genetic correlation among C_1_ traits evaluated at the seedling stage in Scenario (i). However, to estimate genetic correlations for Scenarios (ii) and (iii), we used a two-step procedure. The BLUPs obtained from the first-step analyses described above were deregressed. The deregressed BLUPs for C_0_ and C_1_ were combined into a single dataset and used as response variables in the second step for fitting the multivariate genomic mixed models described for seedling data. This approach estimates a genetic variance-covariance matrix for the traits. The trait variance-covariance matrices were converted to genetic correlation matrices with *cov2cor* function in R.

### Population Structure, Inbreeding, and Genetic Diversity

To assess population structure, genetic diversity, and inbreeding among C_0_ and C_1_, we used 46,760 SNP markers in both C_0_ and C_1_ populations. The markers were filtered to have a minor allele frequency ≥0.01 and formatted as a dosage matrix, with SNP genotypes coded as −1, 0, or +1. The realized genomic relationship matrix (**K**) was constructed with this dosage matrix as input using the *A.mat* function in the rrBLUP package (Endelman, 2011). Principal component analysis (PCA) was conducted on **K** using the *prcomp* function in R. The first two principal components (PCs) were used to visualize population structure. The mean of the diagonals of the matrix **K** is known to be proportional to the inbreeding coefficient (Endelman and Jannink, 2012). Therefore, we used the average of the diagonal elements of **K** as a proxy to measure inbreeding coefficient. On the other hand, we used the average of the off-diagonal elements of **K** as a measure of genetic diversity. These averages were computed separately for the C_0_ and C_1_ populations.

## RESULTS

### Heritability Estimates and Mean GEBVs of C_1_ and C_0_ Clones

Estimates of broad-sense heritability for foliar CBSD scored at 3 and 6 MAP (CBSD severity [CBSD3s] and incidence [CBSD3i] at 3 MAP, and CBSD severity [CBSD6s] and incidence [CBSD6i] at 6 MAP) ranged from 0.28 for CBSD3s to 0.47 for CBSD3i in the C_0_ base population, whereas the estimates of the broad-sense heritability varied from 0.44 for CBSD6s to 0.59 for CBSD6s in the C_1_ base population (Table 1). In general, broad-sense heritability for CBSD root necrosis was higher for the C_1_ (0.45 for CBSDRs and 0.50 for CBSD root incidence at 12-mo harvest [CBSDRi]) than for the C_0_ (0.38 for CBSDRs and 0.37 for CBSDRi) base population. On the other hand, estimates of broad-sense heritability for foliar CBSD ranged from 0.26 for CBSD3s to 0.49 for CBSD3i among selected parents out of C_0_, whereas the broad-sense heritability ranged from 0.52 for CBSD6i to 0.69 for CBSD3i among the selected parents out of C_1_. Overall, the broad-sense heritability estimates of CBSD root necrosis were higher for C_1_ (0.70 for CBSDRs and 0.63 for CBSDRi) selected as parents than for C_0_ (0.29 for CBSDRs and 0.39 for CBSDRi) clones selected as progenitors to generate the C_1_ population.

**Table 1:** Heritability estimates and mean genomic estimated breeding values (GEBVs) for traits measured at the clonal evaluation stage.

	C_0_ base population†		Selected parents out of C_0_‡		C_1_ base population§		Selected parents out of C_1_¶		C_0_ base population mean GEBVs	C_1_ base population GEBVs	C_0_vs C_1_ base populations (*t*test C_0_ vs. C_1_)
Traits#	*H*^2^††	*h*^2^‡‡	*H*^2^	*h*^2^	*H*^2^	*h*^2^	*H*^2^	*h*^2^			
CBSD3s	0.28	0.27	0.26	0.47	0.55	0.57	0.57	0.57	0.04	−0.07	0.11ns§§
CBSD3i	0.47	0.53	0.49	0.72	0.56	0.59	0.69	0.60	1.39	−0.68	2.04ns
CBSD6s	0.32	0.32	0.41	0.59	0.44	0.47	0.65	0.65	0.05	−0.02	0.07***
CBSD6i	0.35	0.36	0.34	0.49	0.59	0.46	0.52	0.68	3.11	−1.53	4.64***
CBSDRs	0.38	0.43	0.29	0.54	0.45	0.06	0.70	0.21	0.09	−0.04	0.13***
CBSDRi	0.37	0.44	0.39	0.65	0.50	0.13	0.63	0.31	2.95	−1.44	4.39***
CMD3s	0.51	0.70	0.49	0.81	0.81	0.59	0.95	0.20	−0.03	0.01	0.04ns
CMD3i	0.60	0.78	0.50	0.81	0.78	0.50	0.59	0.23	−2.16	1.05	3.21**
CMD6s	0.56	0.62	0.61	0.72	0.81	0.55	0.25	0.21	−0.02	0.01	0.03ns
CMD6i	0.50	0.65	0.47	0.77	0.77	0.44	0.08	0.05	−1.53	0.74	2.27*
HI	0.36	0.48	0.40	0.67	0.36	0.18	0.20	0.11	0.01	−0.01	0.02**
RTWT	0.24	0.04	0.06	0.17	0.14	0.00	0.30	0.30	0.02	−0.01	0.03ns
DMC	0.11	0.12	0.07	0.06	0.18	0.08	0.49	0.79	−0.22	0.11	0.33***

*, **, *** Significant at the 0.05, 0.01, and 0.001 probability levels, respectively.

† Initial set of 395 clones for training genomic prediction model (Cycle 0 [C_0_] base population).

‡ Progenitors selected (100 clones) from initial training population to generate genomic selection Cycle 1 (C_1_) population.

§ Second set of 1056 clones referred to as genomic selection Cycle 1 population (C_1_base Population).

¶ Progenitors selected (110 clones) from genomic selection Cycle 1 population to generate Cycle 2.

# CBSD3s, cassava brown streak disease severity assessed at 3 mo after planting; CBSD3i, cassava brown streak disease incidence at 3 mo after planting; CBSD6s, cassava brown streak disease severity assessed at 6 mo after planting; CBSD6i, cassava brown streak disease incidence at 6 mo after planting; CBSDRs, cassava brown streak disease root severity at 12 mo after planting; CBSDRi, cassava brown streak disease root incidence at 12 mo after planting; CMD3s, cassava mosaic disease severity assessed at 3 mo after planting; CMD3i, cassava mosaic disease incidence at 3 mo after planting; CMD6s, cassava mosaic disease severity assessed at 6 mo after planting; CMD6i, cassava mosaic disease incidence at 6 mo after planting; HI, harvest index; RTWT, root weight per plot; DMC, dry matter content.

††Broad-sense heritability estimates for C_0_and C_1_ base populations and their selected progenitors.

‡‡ SNP-based heritability estimates (narrow-sense heritability) for C_0_ and C_1_ base populations and their selected progenitors.

§§ ns, nonsignificant.

Broad-sense heritability for CMD, an important plant health trait, varied from 0.50 for CMD incidence at 6 MAP (CMD6i) to 0.60 for CMD severity at 3 MAP (CMD3s) in the C_0_ base population, whereas the broad-sense heritabilities in C_0_ base population ranged from 0.77 for CMD6i to 0.81 for CMD severity at 6 MAP (CMD6s) for C_1_ (Table 1). Meanwhile, broad-sense heritability ranged from 0.47 for CMD6i to 0.61 for CMD6s, and from 0.08 for CMD6i to 0.95 for CMD3s for selected parents from the C_0_ and C_1_ base populations.

Broad-sense heritability estimates for HI ranged from 0.20 to 0.4 for C_0_ and C_1_ populations, and their selected progenitors (Table 1). The broad-sense heritability estimates were generally low for root DMC (≤0.18) and RTWT (≤0.24), among C_0_, C_1_, and selected parents out of C_0_. In contrast, moderate broad-sense heritability estimates of 0.49 and 0.30 were observed, respectively, for DMC and RTWT for selected C_1_ clones as parents (Table 1).

Estimates of narrow-sense heritability, also referred to as “SNP-based heritability,” for foliar CBSD ranged from 0.27 for CBSD3s to 0.53 for CBSD3i among the C_0_ base population, whereas estimates of narrow-sense heritability varied from 0.46 for CBSD6s to 0.59 for CBSD3i for parents selected from the C_1_ base population (Table 1). For CBSD root necrosis, in general, we observed higher narrow-sense heritability estimates for the C_0_ (0.43 for CBSDRs and 0.44 for CBSDRi) than for the C_1_ (0.06 for CBSDRs and 0.13 for CBSDRi) population. On the other hand, narrow-sense heritability for foliar CBSD ranged from 0.47 for CBSD3s to 0.72 for CBSD3i in C_0_, and from 0.57 for CBSD3s to 0.68 for CBSD6i in C_1_ for selected progenitors (Table 1). Similar to the base populations, estimates of narrow-sense heritability were higher for CBSD root necrosis in C_0_ (0.54 for CBSDRs and 0.65 for CBSDRi) than those in C_1_ (0.21 for CBSDRs and 0.31 for CBSDRi) for selected parents.

For CMD, we recorded relatively high narrow-sense heritability estimates, ranging from 0.62 for CMD6s to 0.78 for CMD incidence at 3 MAP (CMD3i) for the C_0_ base population and from 0.44 for CMD6i to 0.59 for CMD3s for the C_1_ base population. Meanwhile, narrow-sense heritability varied between 0.72 for CMD6s and 0.82 for CMD3i in C_0_, and between 0.05 for CMD6i and 0.23 for CMD3i in C_1_ for selected parents from C_1_. We observed higher SNP-based heritability estimates for HI in the C_0_ base population (*h^2^* = 0.48) and their selected progenitors (*h^2^* = 0.67) than those for the C_1_ base population (*h^2^* = 0.18) and their selected parents (*h^2^* = 0.11). Generally, low (*h^2^* ≤ 0.36) SNP-based heritability estimates were recorded for DMC and RTWT for both the C_0_ and C_1_ populations, except for C_1_ clones selected as parents, where SNP-based heritability estimate was relatively high (*h^2^* = 0.79) for DMC (Table 1).

We compared the average breeding values of C_0_ and C_1_ clones (Table 1). For CBSD in general, the C_1_ clones had better average breeding values (lower disease) than the C_0_ clones. Further, a *t* test of mean differences between C_0_ and C_1_ GEBVs for CBSD6s and CBSDRs, revealed highly significant differences (*P* ≤ 0.001) between the two populations. For CMD, C_0_ clones exhibited better performance (lower disease) than C_1_; however, the mean differences between the GEBVs for the two populations were nonsignificant for both CMD3s and CMD6s (Table 1). A similar trend of nonsignificant average difference in GEBVs was observed for RTWT between C_0_ and C_1_ clones. For DMC, the C_1_ clones had significantly (*P* ≤ 0.001) higher average GEBVs than C_0_ clones (Table 1).

**Fig. 1:**
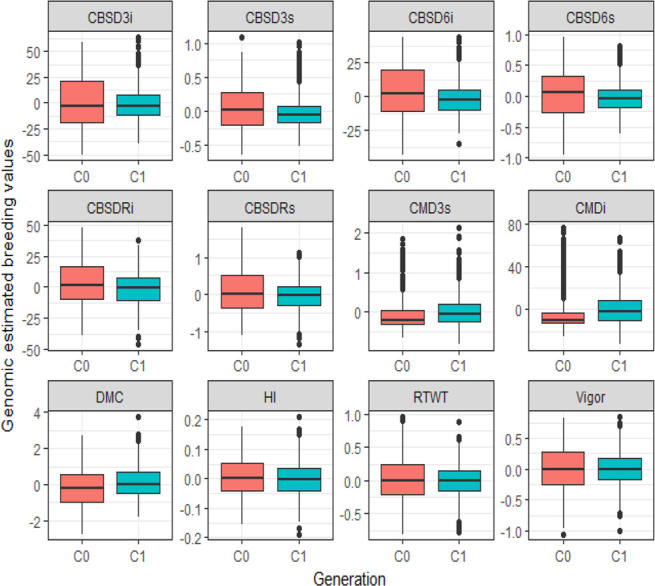
Boxplots showing variability in genomic estimated breeding values for selected plant health (cassava brown streak disease severity assessed at 3 mo after planting [CBSD3s], cassava brown streak disease incidence at 3 mo after planting [CBSD3i], cassava brown streak disease severity assessed at 6 mo after planting [CBSD6s], cassava brown streak disease incidence at 6 mo after planting [CBSD6i], cassava brown streak disease root severity at 12 mo after planting [CBSDRs], cassava brown streak disease root incidence at 12 mo after planting [CBSDRi], cassava mosaic disease severity assessed at 3 mo after planting [CMD3s], and cassava mosaic disease incidence at 3 mo after planting [CMDi]) and agronomic traits (harvest index [HI], root weight per plot [RTWT], and dry matter content [DMC]) of Cycle 0 (C_0_) and Cycle 1 (C_1_) populations evaluated at clonal stage.

Furthermore, using boxplots to compare the variation in GEBVs between C_1_ and C_0_ populations (Fig. 1), a general trend of lower CBSD incidences and severities in C_1_ than in C_0_ was observed for disease assessments at 3, 6, and 12 MAP, with much reduced variability for C_1_ clones. For CMD, HI, and DMC, the level of variation for the GEBVs was relatively similar for C_0_ and C_1_ clones, whereas C_0_ had more variability in their GEBVs than C_1_ for RTWT and plant vigor (Fig. 1).

### Genetic and Phenotypic Correlations among C_0_ and C_1_ Clones

Because root weight for cassava can be estimated reasonably only from clonal-stage evaluations, the seedling evaluation focused on correlations among plant health traits. Highly positive phenotypic and genetic correlations (*r*_p_= 0.88 and *r*_g_ = 0.94, respectively) were recorded between seedling CBSDRs and CBSDRi (Table 2). In general, there were low pair-wise genetic correlations observed among C_1_ seedling traits (plant vigor, CBSD6s, and CMD6s), varying from *r*_g_ = −0.14 to *r*_g_ = 0.24 (Table 2).

**Table 2:** Phenotypic (lower diagonal) and genetic (upper diagonal) correlations among Cycle 1 (C_1_) seedling traits. All the phenotypic and genetic correlations (*r*_p_ and *r*_g_) ≥0.1 in absolute values were significant (*P* ≤ 0.05) at an individual test level.

Traits†	Vigor-ST	CBSD6s-ST	CBSDRs-ST	CBSDRi-ST	CMD6s-ST
Vigor-ST	–	0.04	0.12	0.11	−0.02
CBSD6s-ST	−0.04	–	0.24	0.27	0.10
CBSDRs-ST	0.05	0.03	–	0.94	−0.01
CBSDRi-ST	0.05	0.03	0.88	–	−0.14
CMD6s-ST	−0.04	0.12	−0.03	−0.05	–

† Vigor-ST, seedling plant vigor; CBSD6s-ST, seedling cassava brown streak disease severity assessed at 6 mo after planting (MAP); CBSDRs-ST, seedling cassava brown streak disease root severity assessed at 12 MAP; CBSDRi-ST, seedling cassava brown streak disease root incidence assessed at 12 MAP; CMD6s-ST, seedling cassava mosaic disease severity assessed at 6 MAP.

**Table 3:** Phenotypic correlations for traits measured at seedling and at clonal evaluation stages. All the phenotypic correlations (*r*_p_) ≥0.24 in absolute values were significant (*P* ≤ 0.05) at an individual test level.

Traits†	Vigor-ST	CBSD6s-ST	CBSDRs-ST	CBSDRi-ST	CMD6s-ST
CBSD3s-CT	−0.12	−0.08	0.34	0.29	0.01
CBSD3i-CT	−0.13	−0.08	0.35	0.30	0.02
CBSD6s-CT	−0.08	−0.07	0.26	0.25	0.06
CBSD6i-CT	−0.08	−0.08	0.25	0.24	0.08
CBSDRs-CT	−0.08	0.05	0.39	0.35	−0.11
CBSDRi-CT	−0.09	0.04	0.36	0.36	−0.05
CMD3s-CT	0.03	−0.06	0.01	−0.02	0.05
CMD3i-CT	0.04	−0.07	0.02	−0.01	0.04
CMD6s-CT	0.05	−0.06	0.02	−0.01	0.08
CMD6i-CT	0.07	−0.07	0.01	−0.02	0.11
HI-CT	−0.01	0.01	−0.18	−0.12	0.00
RTWT-CT	0.08	0.03	−0.07	−0.05	0.00
DMC-CT	0.07	−0.04	−0.11	−0.07	0.01
Vigor-CT	0.12	0.00	−0.08	−0.07	0.00

† Vigor-ST, seedling plant vigor; CBSD6s-ST, seedling cassava brown streak disease severity assessed at 6 mo after planting; CBSDRs-ST, seedling cassava brown streak disease root severity at 12 mo after planting; CBSDRi-ST, seedling cassava brown streak disease root incidence at 12 mo after planting; CMD6s-ST, seedling cassava mosaic disease severity assessed at 6 mo after planting; CBSD3s-CT, clonal cassava brown streak disease severity assessed at 3 mo after planting; CBSD3i-CT, clonal cassava brown streak disease incidence at 3 mo after planting; CBSD6s-CT, clonal cassava brown streak disease severity assessed at 6 mo after planting; CBSD6i-CT, clonal cassava brown streak disease incidence at 6 mo after planting; CBSDRs-CT, clonal cassava brown streak disease root severity at 12 mo after planting; CBSDRi-CT, clonal cassava brown streak disease root incidence at 12 mo after planting; CMD3s-CT, clonal cassava mosaic disease severity scored at 3 mo after planting; CMD3i-CT, clonal cassava mosaic disease incidence at 3 mo after planting; CMD6s-CT, clonal cassava mosaic disease severity scored at 6 mo after planting; CMD6i-CT, clonal cassava mosaic disease incidence at 6 mo after planting; HI-CT, clonal harvest index; RTWT-CT, clonal root weight per plot; DMC-CT, clonal dry matter content; Vigor-CT, clonal plant vigor.

**Table 4:** Genetic correlations for traits measured at seedling and at clonal evaluation stage. All the genetic correlations (*r*_g_) ≥0.27 in absolute values were significant (*P* ≤ 0.05) at an individual test level.

Traits†	Vigor-ST	CBSD6s-ST	CBSDRs-ST	CBSDRi-ST
Vigor-CT	0.04	0.01	−0.02	−0.08
CBSD3s-CT	0.05	−0.25	0.23	0.19
CBSD3i-CT	0.08	−0.27	0.30	0.25
CBSD6s-CT	−0.05	−0.14	0.34	0.29
CBSD6i-CT	0.00	−0.19	0.34	0.29
CBSDRs-CT	−0.10	0.31	0.70	0.73
CBSDRi-CT	0.07	0.31	0.77	0.80
CMD3s-CT	0.21	−0.23	0.02	−0.02
CMD3i-CT	0.31	−0.18	−0.05	−0.09
CMD6s-CT	0.42	−0.30	0.05	0.01
CMD6i-CT	0.59	−0.25	−0.02	−0.06
HI-CT	−0.46	0.02	−0.07	−0.12
RTWT-CT	0.04	0.17	−0.11	0.02
DMC-CT	−0.01	0.06	−0.30	−0.31

† Vigor-ST, seedling plant vigor; Vigor-CT, clonal plant vigor; CBSD6s-ST, seedling cassava brown streak disease severity assessed at 6 mo after planting; CBSDRs-ST, seedling cassava brown streak disease root severity at 12 mo after planting; CBSDRi-ST, seedling cassava brown streak disease root incidence at 12 mo after planting; CBSD3s-CT, clonal cassava brown streak disease severity assessed at 3 mo after planting; CBSD3i-CT, clonal cassava brown streak disease incidence at 3 mo after planting; CBSD6s-CT, clonal cassava brown streak disease severity assessed at 6 mo after planting; CBSD6i-CT, clonal cassava brown streak disease incidence at 6 mo after planting; CBSDRs-CT, clonal cassava brown streak disease root severity at 12 mo after planting; CBSDRi-CT, clonal cassava brown streak disease root incidence at 12 mo after planting; CMD3s-CT, clonal cassava mosaic disease severity scored at 3 mo after planting; CMD3i-CT, clonal cassava mosaic disease incidence at 3 mo after planting; CMD6s-CT, clonal cassava mosaic disease severity scored at 6 mo after planting; CMD6i-CT, clonal cassava mosaic disease incidence at 6 mo after planting; HI-CT, clonal harvest index; RTWT-CT, clonal root weight per plot; DMC-CT, clonal dry matter content.

Results for phenotypic correlations between seedling and clonal evaluations are presented in Tables 3 and 4. We recorded moderate to high, positive phenotypic and genetic correlations between CBSDRs scored at seedling stage and other CBSD related-traits, assessed at the clonal stage, the notable of which included (i) CBSD3s (*r*_p_ = 0.34 and *r*_g_ = 0.23) and CBSD3i (*r*_p_ = 0.35 and *r*_g_ = 0.30), and (ii) CBSDRs (*r*_p_ = 0.39 and *r*_g_ = 0.70) and CBSDRi (*r*_p_ = 0.36 and *r*_g_ = 0.77). We observed a similar trend for the phenotypic and genetic correlations between CBSDRi scored at seedling and other CBSD-related traits, with the highest correlation (*r*_g_= 0.80) observed between CBSDRi measured at seedling and CBSDRi at clonal stage (Tables 3 and 4). Unexpectedly, CMD6s with high heritability estimates had low phenotypic correlation (*r*_p_ =0.08) observed between seedling and clonal stages. On the other hand, a negative genetic correlation (*r*_g_ = −0.46) was observed between seedling plant vigor and HI measured at clonal stage.

Finally, we examined phenotypic and genetic correlations among C_0_ and C_1_ traits evaluated at clonal stage (Table 5). We recorded the high phenotypic and genetic correlations ranging from 0.79 to 0.98 between disease severity and incidence scored within the same time point (i.e., at 3, 6, and 12 MAP for both CMD and CBSD). Similarly, we observed high phenotypic and genetic correlations, ranging from 0.51 to 0.95 between foliar disease severities scored at 3 and 6 MAP for both CMD and CBSD. However, there were notably low phenotypic and genetic correlations between CBSD3s and CBSDRs (*r*_p_ = 0.13 and *r*_g_ = −0.12), and between CBSD6s and CBSDRs (*r*_p_ = 0.23 and *r*_g_ = −0.03). Furthermore, we consistently observed negative genetic correlations ranging from −0.32 to −0.17 between disease traits and RTWT, similar to genetic correlations between DMC and CBSDRs (*r*_g_ = −0.64). However, HI had a positive phenotypic and genetic correlations (*r*_g_ = 0.4 and *r*_g_ = 0.54) with root weight per plant. (Table 5). Importantly, all the phenotypic and genetic correlations (*r*_g_) ≥0.2 in absolute values were significant (*P* ≤ 0.05) at an individual test level.

**Table 5:** Phenotypic (lower diagonal) and genetic (upper diagonal) correlations among Cycle 0 (C_0_) and Cycle 1 (C_1_) clonal evaluated traits. The phenotypic and genetic correlations (*r*_p_ and *r*_g_) ≥0.2 in absolute values were significant (*P* ≤ 0.05) at an individual test level.

Traits†	Vigor	CBSD3s	CBSD3i	CBSD6s	CBSD6i	CBSDRs	CBSDRi	CMD3s	CMD3i	CMD6s	CMD6i	HI	RTWT	DMC
Vigor	–	−0.03	−0.06	−0.05	−0.13	0.03	0.04	−0.22	−0.25	−0.21	0.21	−0.02	0.35	0.05
CBSD3s	−0.01	–	0.95	0.83	0.84	−0.12	0.01	−0.04	−0.07	−0.06	−0.06	0.15	−0.20	−0.11
CBSD3i	−0.01	0.79	–	0.90	0.90	−0.15	0.00	−0.08	−0.11	−0.12	−0.10	0.21	−0.17	−0.07
CBSD6s	0.01	0.51	0.52	–	0.95	−0.03	−0.03	−0.28	−0.13	−0.30	−0.29	0.32	−0.21	−0.09
CBSD6i	−0.02	0.49	0.53	0.82	–	0.11	0.11	−0.13	−0.12	−0.12	−0.12	0.26	−0.27	−0.11
CBSDRs	0.01	0.13	0.11	0.23	0.17	–	0.95	−0.22	−0.19	−0.15	−0.20	−0.37	−0.20	−0.64
CBSDRi	0.01	0.15	0.13	0.24	0.18	0.92	–	−0.31	−0.28	−0.25	−0.28	−0.26	−0.18	−0.58
CMD3s	−0.11	−0.06	−0.11	−0.12	−0.09	−0.10	−0.10	–	0.98	0.95	0.95	−0.46	−0.29	0.01
CMD3i	−0.10	−0.09	−0.12	−0.14	−0.11	−0.10	−0.10	0.91	–	0.94	0.95	0.44	−0.32	0.00
CMD6s	−0.13	0.02	−0.05	−0.03	0.00	−0.05	−0.06	0.70	0.68	–	0.97	−0.43	−0.31	−0.05
CMD6i	−0.13	−0.01	−0.05	−0.05	−0.02	−0.07	−0.06	0.70	0.71	0.90	–	−0.41	−0.24	−0.05
HI	0.17	0.00	0.02	0.01	0.03	−0.24	−0.22	−0.09	−0.09	−0.13	−0.12	–	0.41	0.23
RTWT	0.21	−0.03	0.01	0.00	0.01	−0.22	−0.22	−0.09	−0.08	−0.09	−0.08	0.54	–	0.19
DMC	0.22	−0.17	−0.15	−0.28	−0.24	−0.39	−0.36	0.04	0.09	−0.07	−0.04	0.42	0.16	–

† Vigor, plant vigor scored at 3 mo after planting; CBSD3s, cassava brown streak disease severity scored at 3 mo after planting; CBSD3i, cassava brown streak disease incidence at 3 mo after planting; CBSD6s, cassava brown streak disease severity scored at 6 mo after planting; CBSD6i, cassava brown streak disease incidence at 6 mo after planting; CBSDRs, cassava brown streak disease root severity at 12 mo after planting; CBSDRi, cassava brown streak disease root incidence at 12 mo after planting; CMD3s, cassava mosaic disease scored at 3 mo after planting; CMD3i, cassava mosaic disease incidence at 3 mo after planting; CMD6s, cassava mosaic disease severity scored at 6 mo after planting; CMD6i, cassava mosaic disease incidence at 6 mo after planting; HI, harvest index; RTWT, root weight per plot; DMC, dry matter content.

### Population Structure and Level of Inbreeding in C_0_ and C_1_ Clones

**Fig. 2:**
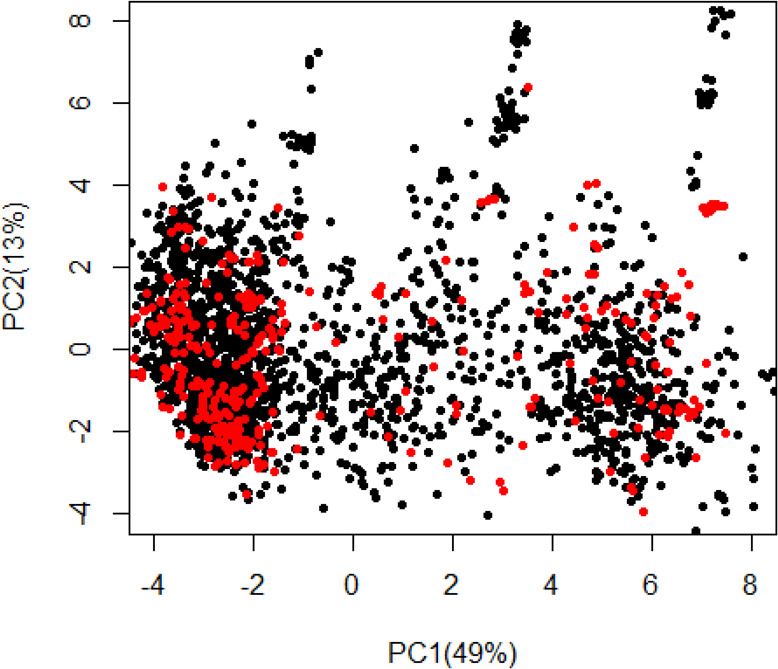
Population structure from a plot of Eigenvalues of Principal Component 1 (PC1) against Principal Component 2 (PC2), using realized genomic relationship matrix for Cycle 0 (C_0_) and Cycle 1 (C_1_) populations. The C0 population (red) comprised 395 individuals, and C_1_ (black) comprised 1056 clones. The population structure was estimated from kinship matrix constructed, using 46,760 SNP markers, filtered at minor allele frequency ≥0.01.

**Fig. 3:**
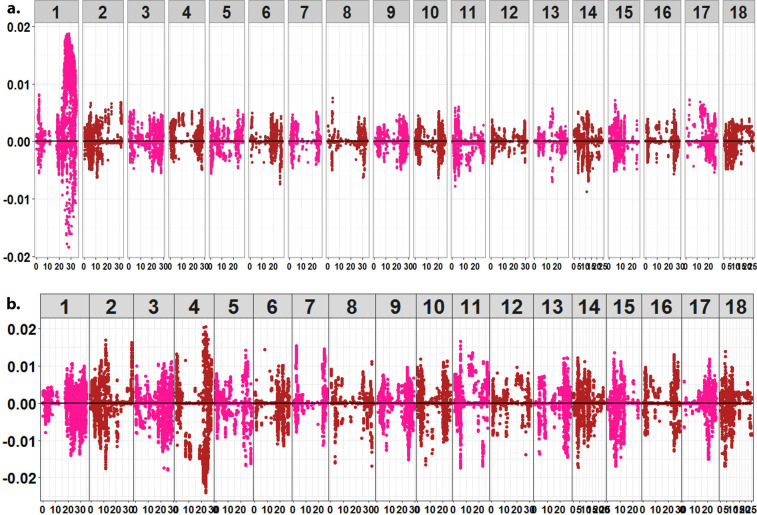
A plot of the loadings (Eigenvector coefficients) for each marker on (a) Principal Component 1 (PC1) and (b) Principal Component 2 (PC2) against marker position along the 18 cassava chromosomes. Markers affecting PC1 most strongly loaded on the first chromosome. Markers explaining the largest variation for PC2 loaded on chromosome 4

**Fig. 4:**
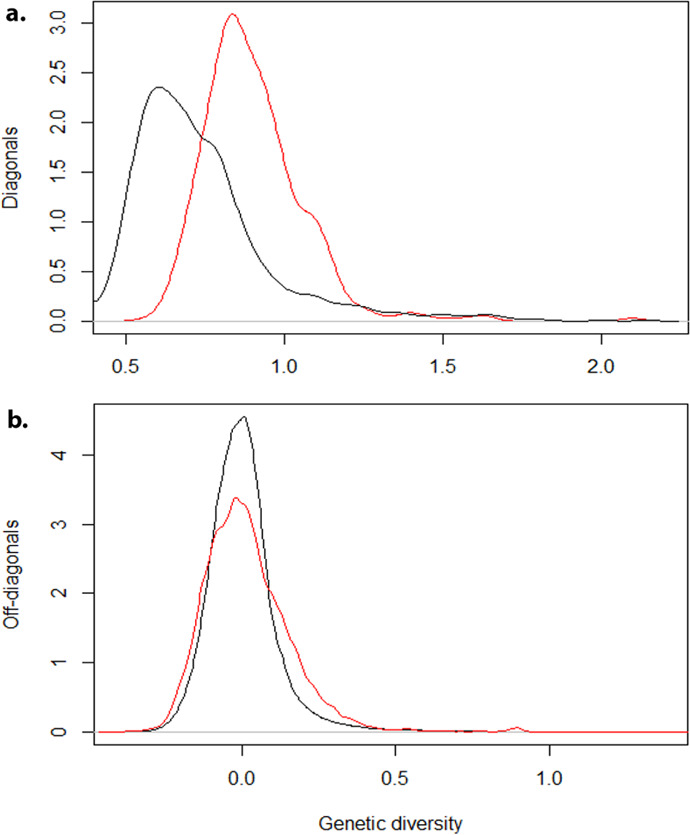
Density plots generated from (a) the diagonal elements and (b) the off-diagonal elements of realized genomic relationship matrix, as measurements of inbreeding levels for Cycle 0 (C_0_, red) and Cycle 1 (C_1_, black) populations. In Panel a, the density plots indicated that there was less inbreeding in C_1_ than in C_0_ clones, based on estimates from 46,760 single nucleotide polymorphism (SNP) markers. In Panel b, both C_0_ and C_1_ had similar diversity estimated by 46,760 SNP markers, implying that the original diversity in the C0 base population was captured by the selected parents that generated the C_1_ population.

Based on PCA, there was no clear genetic differentiation between C_0_ and C_1_ populations. Indeed, majority of the total genetic variation (49%) in C_0_ and C_1_ populations was explained by the first PC, with 13% attributed to PC2 (Fig. 2). Further plots of the loadings (eigenvector coefficients) for each marker on PC1 and PC2 against marker position along the 18 cassava chromosomes revealed that markers affecting PC1 and PC2 most strongly were on the first and fourth chromosome, respectively (Fig. 3a and 3b). The means of the diagonals of the kinship matrix, which is proportional to one plus the inbreeding coefficient (1 + *F*), were 0.904 in C_0_ and 0.708 for C_1_ (Fig 4a). Density plots of the off-diagonal elements of the kinship matrix indicated that the degree of variability in relatedness was similar in the C_0_ and C_1_ (Fig. 4b).

## DISCUSSION

In this paper, we highlight progress that has been made towards increasing the productivity of cassava for the benefit of communities that depend on it. Notable production obstacles addressed include susceptibility to CMD and CBSD, and low yields of both fresh root and dry matter. In response to these challenges, the cassava breeding program based at NaCRRI initiated efforts to implement GS to accelerate the breeding cycle of cassava (Wolfe et al., 2016, 2017). This paper, therefore, aimed at examining genetic variability and correlations in GS populations (C_0_ and C_1_), as well as their respective selected progenitors. Our primary traits of focus included diseases (CMD and CBSD) and yield components (DMC, HI, and RTWT).

### Heritability Estimates and Mean GEBVs of C_1_ and C_0_ Clones

The relatively high heritability for CMD has already facilitated identification of resistant cultivars from various breeding programs through phenotypic selection (Thresh and Cooter, 2005; Egesi et al., 2007; Kawuki et al., 2016). In general, phenotypic selection would be cost effective to select for highly heritable traits, such as CMD. However, to produce desirable cultivars, our breeding program seeks to improve a number of traits, alongside CMD, many of which are quantitative traits, such as yield and CBSD resistance with low to moderate heritability. It is mainly for these yield and CBSD resistance traits that we employ GS, using a selection index to improve all focal traits simultaneously.

Moderate to high estimates of broad-sense heritability for foliar and root severities were registered for CBSD (ranging from 0.26 to 0.70) in both populations studied here. The broad-sense heritability estimates for CBSD in the present study were comparable with heritability estimates previously reported (Kayondo et al., 2018), ranging from 0.25 for CBSDRs to 0.61 for CBSD3s, from the genome-wide association study involving ~1300 clones evaluated across five sites in Uganda. These moderate to high broad-sense heritability estimates for CBSD indicated that selection efficiency either through conventional or GS would be high enough to achieve the desired genetic gains.

Fresh root weight and DMC, which are key traits for cassava production, had the lowest broad-sense heritability estimates. Root weight as a measurement of yield is known to be polygenic and influenced by environment (Fukuda et al., 2002). According to Barandica et al. (2016), measurements of yield and its components are best estimated at later stages of the cultivar selection pipelines, when plot sizes are larger than those in the current study. It is partly for this reason that HI has been proposed as an indirect measurement of yield (Kawano et al., 1987). Indeed, in our study, HI had higher broad-sense heritability estimates than root weight, ranging between 0.2 and 0.44 compared with 0.14 and 0.30 for fresh RTWT. Thus, selecting on HI could be complementary to direct selection on fresh root yield, particularly at earlier stages of evaluation. The breeding program can then place heavier emphasis directly on root weight during later stages, when plot sizes are large enough for accurate assessment.

Broad-sense heritability estimates for DMC were particularly low (0.00–0.18), except for clones selected as parents of C_1_. Clearly, our broad-sense heritability estimates for DMC were lower than the heritability estimate of 0.46 reported by Wolfe et al. (2017). In part, the low heritability estimates of DMC in the present study could be attributed to the effect of CBSD on the DMC. A previous study of CBSD effect on DMC reported significant differences between healthy roots and those with necrotic symptoms of CBSD (Nuwamanya et al., 2015). Often, infected roots with CBSD become necrotic; necrosis limits the quantity and quality of root samples used for DMC estimation via specific gravity method. Principally, specific gravity proposed by Kawano et al. (1987) uses 3- to 5-kg samples; in some cases, we used weights of <3 kg for estimation of DMC. Furthermore, the adverse effect of CBSDRs on DMC is evident from the high negative genetic correlation (*r* = −0.64) observed in this study.

The SNP-based heritability estimates were generally lower than broad-sense heritability for most traits evaluated for C_1_ clones. The precise reason for this seeming discrepancy between broad- and narrow-sense heritability estimates is not known. However, it has been shown both theoretically and with simulations on real data that one reason for the bias in heritability estimation using markers is variation in the amount of linkage disequilibrium (LD) between the markers and the causal loci. If the most important loci are in LD with many more markers than lesser causal loci, then the SNP-based heritability estimates can be upwardly biased. In contrast, if important causal loci are under-tagged by markers, the heritability estimates can be downwardly biased (Speed et al., 2012; de los Campos et al., 2015).

Thus, it is possible that high SNP-based heritability estimates observed for traits, such as CMD3s (*h^2^* = 0.81) in C_0_ and DMC (*h^2^* = 0.79) in C_1_ selected as parents, could be attributed to uneven LD between SNPs. Variation in LD has been previously reported in cultivated cassava (Bredeson et al., 2016), with notably low recombination rates observed in regions of introgression from a wild relative (*Manihot glazovii* Allem) on chromosomes 1 and 4. These variations in LD patterns across the genome could therefore have led to over- or underestimation of SNP-based heritability observed in the present study.

### Estimates of Phenotypic and Genetic Correlations among Traits

Elsewhere, selection of parents at seedling stage for recombination has been reported to drastically shorten the breeding cycle of cassava for highly heritable traits (Ceballos et al., 2013). In this study, we observed a high genetic correlation (*r*_g_ = 0.70) between seedling and clonal CBSD root severities, suggesting the usefulness of seedling data in parental selection for recombination, training GS models, or in selection of clones for further evaluation, targeting cultivar release. Furthermore, CBSD has been reported to spread rapidly in the last two decades in Africa (Hillocks et al., 2002; Alicai et al., 2007; Legg et al., 2011; Mulimbi et al., 2012) to cover countries other than the original CBSD-endemic coastal region of eastern Africa. The high genetic correlation observed between CBSD on seedlings and clonal stages would leverage preemptive breeding for CBSD in West Africa through evaluation of botanical seeds from West Africa in CBSD endemic areas.

We did not expect such low phenotypic correlation as observed for CMD assessments at seedling and clonal evaluation stages (*r*_p_ ≤ 0.12), as it is a trait known to be highly heritable (Wolfe et al., 2016). Often, seedlings that are heavily infected with CMD (severity scores > 3) do not get cloned. Because we discarded seedlings that were highly infected with CMD at 6 MAP and did not collect leaf samples for DNA extraction from those, the overall genetic variance for CMD was decreased among the selected seedlings advanced to clonal stage. Also, some of the symptomless seedlings eventually succumbed to CMD at clonal evaluation. Scenarios of reemergence of latent *Cassava mosaic virus* have been observed previously from plants coinfected by two isolates, interacting in an antagonistic manner (Karthikeyan et al., 2016). One possible explanation would be that some seedlings had latent infection and eventually expressed CMD symptoms at clonal evaluation. A study by Ogbe et al. (2003) reported a low correlation between CMD symptom expression and virus titer, implying the some genotypes harbored a CMD-causing virus without necessarily showing disease symptoms until the virus population within the host plant had reached certain threshold to cause visible symptoms. Those phenomena could explain the low phenotypic correlation observed for seedling and clonal CMD datasets.

**Fig. 5:**
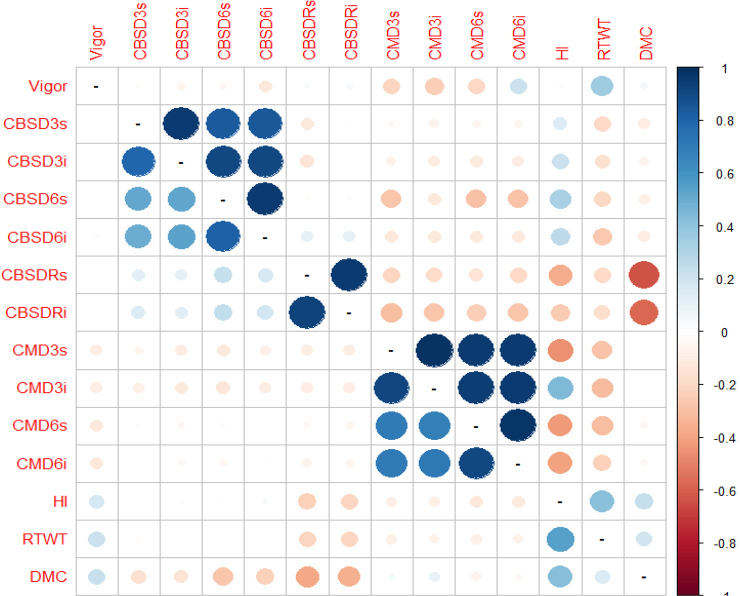
Phenotypic (lower diagonal) and genetic (upper diagonal) correlations among traits (cassava brown streak disease severity assessed at 3 mo after planting [CBSD3s], cassava brown streak disease incidence at 3 mo after planting [CBSD3i], cassava brown streak disease severity assessed at 6 mo after planting [CBSD6s], cassava brown streak disease incidence at 6 mo after planting [CBSD6i], cassava brown streak disease root severity at 12 mo after planting [CBSDRs], cassava brown streak disease root incidence at 12 mo after planting [CBSDRi], cassava mosaic disease severity assessed at 3 mo after planting [CMD3s], cassava mosaic disease incidence at 3 mo after planting [CMDi], harvest index [HI], root weight per plot [RTWT], and dry matter content [DMC]) evaluated at clonal stage for combined Cycle 0 (C_0_) and Cycle 1 (C_1_) populations.

Examination of phenotypic and genetic correlations between disease severity and incidence for CBSD and CMD measured at 3 and 6 MAP revealed high positive genetic correlations (*r*_g_ ≥ 0.83, Table 5, Fig. 5). Similar results were previously reported by Rwegasira and Rey (2012), where a phenotypic correlation of up to 0.98 was reported between foliar disease severity scored at 3 MAP and 6 MAP of CBSD. While Rwegasira and Rey (2012) reported only phenotypic correlations for foliar CBSD severity scored at 3 MAP and 6 MAP, we report both phenotypic and genetic correlations for CBSD severity scored at 3 MAP and 6 MAP. In addition, we scored the foliar disease incidence at both 3 MAP and 6 MAP. These high correlations imply that data collected for disease incidence, especially on foliar plant health status, would be sufficient and recommended, because scoring disease incidence is quicker and less subjective (absence or presence) than scoring disease severity on a wide scale (1–5). In contrast, there were very low phenotypic and genetic correlations between foliar CBSD symptoms (at 3 or 6 MAP) and CBSD root necrosis symptoms (at 12 MAP), which is consistent with earlier studies (Rwegasira and Rey, 2012).

Indeed, Nzuki et al. (2017) recently reported two QTL (located on chromosomes 1 and 12) to be significantly associated with CBSD root necrosis, and four other QTL (located on chromosomes 2, 4, 6, and 17) controlling foliar CBSD severity, suggesting some degree of independence in the genetic control of CBSD resistance. The high genetic correlation between foliar CBSD3s and CBSD6s in the current study implies the possibility of single and effective assessment of CBSD foliar symptoms at 6 MAP, permitting more efficient use of resources. Meanwhile, the high and positive phenotypic (*r*_p_ = 0.56) and genetic correlation (*r*_g_ = 0.41) between HI and root weight with the large number of clones evaluated in the present study agree with the results of previous studies conducted in other breeding populations (Ojulong et al., 2010; Akinbo et al., 2012), suggesting that HI could be used as a complementary trait for RTWT to select for fresh root yield, particularly at early stages of selection, when a large number of clones are evaluated in smaller plots.

### Population Structure and Level of Inbreeding in C_0_ and C_1_ Clones

We did not observe a distinct differentiation between C_0_ and C_1_ populations, which indicates that little or no genetic diversity was lost because of selection using genomic predicted breeding values. Elsewhere, strong population stratification between the training set and the selection candidates has been reported to negatively affect genomic prediction accuracies in oat (*Avena sativa* L.) and rice (Asoro et al., 2011; Grenier et al., 2016). In the present study, the absence of population structure suggests the appropriateness of using C_0_ as a training population for genomic predictions of C_1_ and subsequent selection of parents using GEBVs. However, PC1 explained 49% of total genetic variation, suggesting a subpopulation structure, when considering the two populations jointly. Further examination of PC1 and PC2 marker scores across the 18 chromosomes of C_0_ and C_1_ populations revealed that markers with the strongest effects loaded on chromosomes 1 and 4 for PC1 and PC2, respectively (Fig. 3a and 3b). This finding corroborates with Bredeson et al. (2016), where chromosomes 1 and 4 were found to harbor large pieces of haplotype introgression from *Manihot glazovii* in many of the tropical *Manihot esculenta* (TME) and tropical *Manihot* selection clones. These introgressions are believed to have occurred at the time of pioneer CMD and CBSD breeding at the Amani breeding station in Tanzania (Storey and Nichols, 1938). It also suffices to note that a significant number of the C_0_ clones share ancestry with the *M. esculenta* and/or tropical *Manihot* selection lines that were introduced in Uganda between 1990s and early 2000s.

For both the C_0_ and C_1_ populations, the average of diagonal elements of **K**, as a measure of inbreeding coefficient (1 + *F*) based on markers, was 0.904 and 0.708, respectively. These values should be interpreted to mean that the clones were less inbred than might be expected on the basis of the marker allele frequencies (i.e., the heterozygous marker genotypes were more frequent than expected under Hardy–Weinberg equilibrium). Cassava is known to suffer from inbreeding depression (Rojas et al., 2009; Kawuki et al., 2016; Ramu et al., 2017). Thus, selection among clones in establishing the C_0_ population might have removed clones that were inbred. For the C_1_ population, the priority given to between-, rather than within-, cluster crosses could also be expected to generate above average heterozygosity. Comparison of inbreeding levels in C_0_ and C_1_ populations indicated less inbreeding in C_1_ population than in C_0_. As indicated, we think the crossing strategy we designed accounts for this observation. Evident in the current study was the better average performance of the C_1_ population than the C_0_ population, an indication of overall genetic progress for most traits, which could be a result of less average inbreeding exhibited by C_1_, as indicated by comparing the mean diagonals of the kinship matrix.

## CONCLUSION

From the datasets presented, three major conclusions are drawn: first, seedling evaluation for CBSD, within limits, predicts CBSD clonal performance. This finding justifies selection for CBSD at the seedling stage; for this, the use of both incidence and average root severity can suffice. Second, we observed moderate to high genetic correlations between foliar assessments made for CBSD and CMD at 3 and 6 MAP. This finding justifies a single evaluation done at 6 mo; such a strategy could significantly reduce costs associated with data collection in multi-location trials. Third, selection on GEBVs did not erode the original genetic diversity and resulted in genetic progress for most traits as advances were made from C_0_ to C_1_. Given these results, we do not expect GS to cause rapid inbreeding as breeding populations are moved from one cycle of GS to the next.

## Supplemental Materials Available

Supplemental Table S1. Summary of parents used to generate initial GS populations (Cycle 0).

Supplemental Table S2. Phenotype used to calculate heritability, genomic estimated breeding values, and phenotypic and genetic correlations for seedling and clonal evaluated traits, and correlations among traits evaluated at clonal stage for C_0_ and C_1_ populations.

Supplemental Table S3. Phenotypes used for estimating phenotypic and genetic correlations among traits measured at the seedling stage, and for correlations of seedling vs. clonal measured traits among C_1_ individuals.
